# Skin care habits of patients with eczema at Kiambu Level 5 Hospital, Kiambu county, Kenya: A retrospective cross-sectional study

**DOI:** 10.1016/j.jdin.2024.04.011

**Published:** 2024-05-07

**Authors:** Winnie Njenga, Magoma Mwancha-Kwasa, Kinara Fossa, Prabhjot Kaur Juttla, Fredrick Kimani, Winfred Mwikya, Francis Makokha, Hannah Wanyika

**Affiliations:** a Department of Health, County Government of Kiambu, Kiambu, Kiambu County, Kenya; b Central Province Response Integration Strengthening and Sustainability Project (CRISSP), University of Nairobi, Nairobi, Kenya; c Faculty of Health Sciences, School of Medicine, University of Nairobi, Nairobi, Kenya; d Directorate of Research and Renovations, Mount Kenya University, Thika, Kiambu County, Kenya; e Dermatology Department, Kenyatta National Hospital, Nairobi, Kenya

**Keywords:** eczema, geospatial mapping, Kenya, prevalence, skincare

## Abstract

**Background:**

There is a dearth of data on eczema from the African continent despite the purported increasing burden.

**Objectives:**

To describe the prevalence of eczema at Kiambu Level 5 Hospital and patient skincare.

**Methods:**

A descriptive retrospective cross-sectional research design was performed describing the period between 2016 and 2020. Data analysis was done using STATA Version 13. Geospatial mapping of patient residence was also conducted.

**Results:**

Eczema was the most common skin condition diagnosed with a prevalence of 25.5%. Majority of the patients favored the use of non-conventional products such as bar soap and milking jelly as routine skincare products. Geospatial mapping demonstrated a higher prevalence of eczema in the urban areas and also revealed access to a specialist as a contributor to health-seeking behavior.

**Limitations:**

Subtypes of eczema were not described and this data only reflects 1 facility serving an entire county.

**Conclusion:**

The prevalence of eczema in Kiambu Level 5 Hospital mirrors the increasing global burden of eczema. Urban environments have a higher density of eczema in Kiambu. More research is needed to decipher the impact of the preferred non-conventional skin care products on eczema.

**Capsule Summary**

• The high global eczema prevalence is replicated in Africa, specifically Kenya as exhibited by our paper.
• The study provides data from Kenya, showcasing eczema prevalence in a county clinic and shedding light on unconventional skincare practices and residential environments of patients with eczema

## Introduction

Eczema, also known as dermatitis, is a common skin condition.^1^ This condition is diagnosed clinically based on clinical features which range from erythema, edema, vesiculation, and weeping in acute cases, to lichenification and pigment changes in the more chronic forms.^1^^,^^2^ The Global Burden of Disease report ranked eczema 15th of non-fatal disorders and first for skin diseases measured in disability-adjusted life years.^3^^,^^4^

An international study by Langan et al showed that the prevalence of eczema has increased by 0.98% per decade in adolescents and 1.21% in children.^4^ However, the African continent is under-represented in literature, despite being occupied by a population of more than 1.2 billion people and the prevalence of eczema appearing to increase.^1^ The International Study of Asthma and Allergies in Childhood study demonstrated that between 1995 and 2002, the prevalence of eczema in Kenyan adolescents doubled.^1^^,^^5^ In contrast, in the same time period, they established that the prevalence of eczema in Nigeria reduced. A 2014 study found a remarkable prevalence of 43% in Namibians aged 15 to 30 years.^6^

The International Study of Asthma and Allergies in Childhood attributed the increasing prevalence of eczema in low-and middle-income countries to increasing industrialization, not allergies.^3^^,^^5^ It has been found that urban areas with more pollution record higher prevalence of eczema, suggesting that environmental factors play a pivotal role in the increasing prevalence.^6^ Furthermore, the International Study of Asthma and Allergies in Childhood study points out unique factors in the Sub-Saharan Africa population such as the application of natural butters and plants as skincare, as well as sanitation concerns, which might influence the development of eczema.^7^ Frequent washing with soap has been identified as a contributing factor to the development of eczema in Ghana.^8^

The key feature of eczema is a recurrent itchy rash which tends to have a chronic nature.^6^ The typical age of onset for eczema is in the first year of life, with many cases resolving over time.^6^^,^^9^ A population-based study in the United Kingdom observed the peak incidence of eczema at less than 1 year of age and older than 80 years of age.^9^ A slight female predominance in prevalence of eczema has been noted,^10^ and eczema has been shown to occur concurrently with other skin conditions, for example, seborrheic dermatitis.^11^ The main aim of treatment is to control symptoms and to restore the skin barrier.^12^ The first-line of treating eczema is regular and liberal application of moisturizers, along with use of gentle soaps.^1^^,^^12^ This highlights the importance of skin care habits in patients who suffer from eczema.

In Kenya, the use of petrolatum products is quite common. Local newspapers have reported on the use of Milking Jelly as a skin care product.^13^^,^^14^ This product is designed for application on the teats of cows during milking.^14^ It is an unrefined petrolatum product whose effects on human skin are undocumented. Its availability in supermarkets, even those in cities where cows are not kept, predicates its popularity as a moisturizer.^13^ It may be preferred to conventional petrolatum products made for human use as it retails for about half the cost.^13^

In our setting, accessing data on dermatological conditions including eczema, is an onerous task because well-designed tools to collect this data do not exist. However, there exists a tool in the Kenya Health Information System for aggregate data that documents all skin diseases. The first objective of this paper is to evaluate the prevalence of eczema in the Dermatology Clinic at Kiambu Level 5 Hospital (KL5H). Secondly, it is to characterize the skin care practices of the patients as well as the environment which they inhabit.

## Methods

### Study design and sampling

The study design was a descriptive retrospective cross-sectional study that scrutinized the records of patients attending the Dermatology Clinic at KL5H from 2016 to 2020.

### Study site

The study area was KL5H, located in Kiambu County which borders Nairobi, the capital city.

Kiambu County has an almost-even mix of urban and rural population. It has 12 subcounties, including Kiambu subcounty, where KL5H is located (longitude 36.83098 latitude −1.17209). The total county population was 2,417,735. The number of patients with skin diseases in 2019 was 187,334. The minimum prevalence of dermatological conditions in Kiambu County was 77.5 per 1000 in 2019.

### Study population

The total number of patients seen in the dermatology clinic (*N* = 1183) was determined using the facility's Dermatology Clinic Register.

### Study sample

From the total 1183 patients reporting to the clinic during the study period, 302 had a diagnosis of eczema. Of these, a random sample of 136 eczema patients was obtained.

The inclusion criteria was patients with a recurrent itchy rash, with a background of dry skin. The exclusion criteria was a rash that did not reach the clinical diagnosis of eczema. Included patient files were perused and relevant data abstracted. Variables collected were: patient age, sex, skin care habits (soap and moisturizer), and residence.

### Data analysis

Analysis was conducted using StataCorp. 2013 (*Stata Statistical Software: Release 13*). The characteristics of the participants were summarized in frequencies and proportions. The Geographic Information System analysis and mapping was done in Stata SPMap package using the patient residences.

### Ethical considerations

The study adhered to the World Medical Association's Code of Ethics (Declaration of Helsinki), ensuring confidentiality by omitting personal identifiers throughout data collection, entry, and analysis.

## Results

The prevalence of eczema in the patients seen at the dermatology clinic at KL5H was (302/1183) which translates to 25.5%

From April 2016 to December 2020, 1183 patients were seen in the clinic. Table I shows that 60.6% of the patients seen were female, giving a female-to-male ratio of 3:1. The largest age group of dermatology patients was <5 years of age.

**Table I:** The sociodemographic characteristics of the patients seen at the Kiambu Level 5 Hospital dermatologic clinic between April 2016 and December 2020

	Percentage (%)	Number (*N* = 1183)
Sex		
Female	60.6	717
Male	39.3	465
Missing	0.1	1
Age, y		
<5	20.8	246
6-10	12.6	149
11-20	15.9	188
21-30	19.7	233
31-40	12.1	143
41-50	7.2	85
51-60	5.2	61
>60	6.6	78

**Fig 1:**
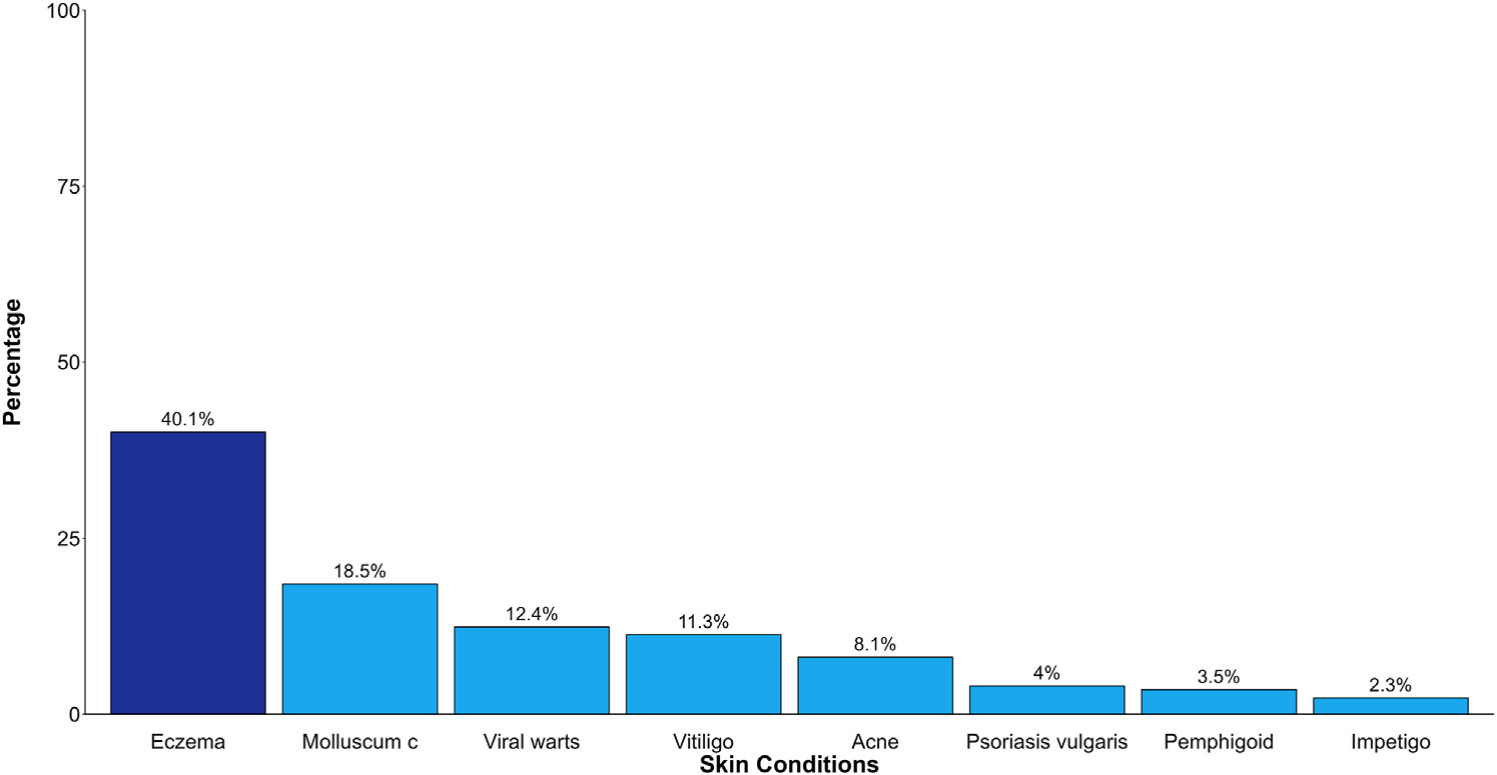
Skin conditions diagnosed at the Dermatology Clinic of Kiambu Level 5 Hospital.

The common conditions diagnosed in the dermatology clinic at KL5H are shown on Fig 1.

Majority of the patients were female (63.2%) with a female-to-male ratio of 1.7:1. The age distribution of patients with eczema displayed the highest prevalence in the age group <5 years with a prevalence of 22.8%. It was closely followed by age group 6-10 years (17.6%). The data also shows a high prevalence in adulthood notably at 21 to 30 years (13.2%). 40.4% of the patients were less than 10 years old. These findings are shown in Table II.

**Table II:** The sociodemographic characteristics of sampled (*n* = 136) patients diagnosed with eczema at the Kiambu Level 5 Hospital dermatologic clinic between April 2016 and December 2020

	Percentage (%)	Number (*n* = 136)
Sex		
Female	63.2	86
Male	36.8	50
Age, y		
<5	22.8	31
6-10	17.6	24
11-20	7.4	10
21-30	13.2	18
31-40	8.8	12
41- 50	12.5	17
51-60	8.1	11
>60	8.1	11

The soaps and moisturizers used by the patients at first contact with the Dermatology clinic were documented as summarized on Table III. The most preferred soap was multipurpose bar soap (38%) while the most preferred product was milking jelly (18.4%). Dermatological (emollient) creams were used by 13.1% and petroleum jelly was used by 11.7%. Together, petrolatum products were the majority favored moisturizers.

**Table III:** An overview of the soaps and moisturizers used by patients diagnosed with eczema upon their index visit to the Kiambu Level 5 Hospital Dermatology Clinic

	Percentage (%)	Number (*n* = 136)
Soaps		
Bar soap	38.2	52
Prescribed soap	8.0	11
Toilet soap	7.4	10
Medicated soap	5.9	8
No soap	5.9	8
Herbal soap	0.8	1
Not indicated	33.8	46
Moisturizers		
Milking jelly	19.0	26
Dermatological creams	13.1	18
Petroleum jelly	11.7	16
None	10.9	15
Lotions	3.6	5
Glycerine	2.2	3
Steroid creams	0.7	1
Vegetable cooking oil	0.7	1
Data not available	38.0	52

**Fig 2:**
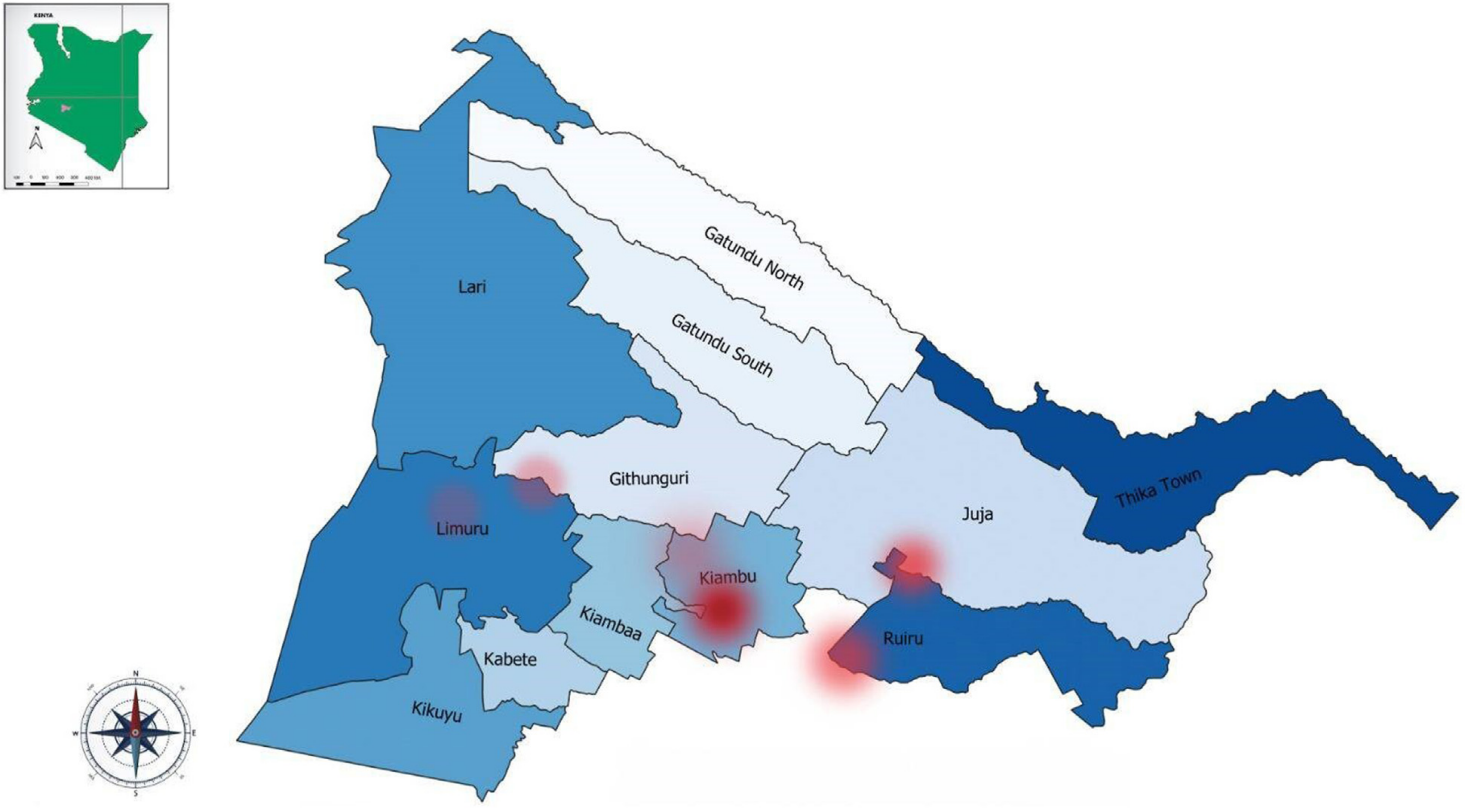
Eczema temporospatial distribution in Kiambu County, Kenya.

Fig 2 illustrates the residence of the eczema patients diagnosed in the dermatology clinic. A hotspot cluster was observed in Kiambu town subcounty, and a few smaller clusters were observed in Roysambu, Kiambaa, and Githunguri subcounties. Majority of the eczema patients come from Kiambu town subcounty and adjacent subcounties, as shown by the color gradient.

A third (34.1%) of eczema patients had been diagnosed with seborrheic dermatitis in the course of their dermatology follow up. This suggests that atopic dermatitis could be a continuum of what presents as seborrheic dermatitis in infancy or possibly, a precursor. An interesting finding was the coexistence of acne in 4 patients (2.9%). Only 2 patients had concurrent vitiligo.

## Discussion

In our experience, the lack of a standardized, properly classified, data collection tool in Kenya Health Information System, presented a challenge in data collection. The Kenya Health Information System classifies all dermatological conditions as “diseases of the skin” at the facility, county, and national levels. It is therefore not possible to abstract data on specific diagnoses. The manual file recording system in the facilities also poses a challenge as some files are unretrievable due to storage and retrieval difficulties. This led to the use of a sampling system in this study as opposed to a census which should be the preferred option.

The prevalence of eczema in the patients seen at the dermatology clinic in the current study was 25.5%. A hospital-based, cross-sectional study conducted in southern Ethiopia found a similar prevalence of eczematous dermatitis at 23.9%.^15^ The latter study was restricted to children seen in the dermatology clinic, while the former study took both pediatric and adult age groups into account. The proportions are similar but different factors may have influenced them such as, study durations and designs, geographical location, socio-economic status, and the environment (urban, rural and community-based populations).^15^

The most common diagnosis in the KL5H dermatology clinic was eczema. This supports global trends in the burden of eczema.^3^^,^^4^^,^^6^ The ratio of female to male patients with eczema in the KL5H Dermatology clinic was about 1.7:1. A community-based study conducted in Angola, found a 1:1 female to male eczema prevalence.^16^ This study evaluated an urban population and observed 6-7-year-old children. However, hospital-based studies in Nigeria and Egypt found similar female to male ratios to our study that were conducted in metropolitan areas.^17^^,^^18^ The gender ratio may also be influenced by the health-seeking behavior of men.^19^

Patients under 5 years of age were the most affected by eczema. This was followed by the 6-10 years age group with a prevalence of 17.6%. This supports the global trends of eczema in which children are significantly affected.^3^ A study conducted in Tanzania found that 50% of all skin diseases affected children under the age of 15 years old.^20^ Another hospital-based study conducted in southern Ethiopia found a majority of their patients were between 5 and 10 years old (44.9%).^15^ Our study also demonstrates a significant burden in adults. The most affected age group is the 21-30 years one with 13.2%. A prospective approach might capture the trend of eczema in adulthood better, given that we cannot deduce the age of onset of eczema especially in adult patients.

Our study found the presence of acneiform rashes in a few patients who were diagnosed with eczema. Usually, acne and eczema do not coexist. However, acneiform rashes can arise as a side effect of using corticosteroids used to manage eczema.^21^ It is also important to note that the use of petrolatum products, which is common in our set up, can also contribute to the development of acneiform rashes.^22^ In the current study, 1 patient was routinely using a topical corticosteroid. This points to misuse of topical corticosteroids and warrants further investigation. In contrast to the findings of our study, the most common comorbidity of eczema was secondary bacterial infections.^23^ In our setting, patients will be seen in the outpatient prior to a dermatology appointment where antimicrobials will usually be prescribed if needed. This could reduce the apparent number of infections diagnosed in patients in our clinic. This could be explained by the fact that patients who are seen in the Dermatology Clinic are first attended to by primary care doctors in outpatient who likely would have treated infections if present.

A third of patients with eczema in our study had been diagnosed with seborrheic dermatitis in the course of their follow up. This could suggest that seborrheic dermatitis predisposes individuals to development of eczema later in life, or that they coexist in our setting. A skin barrier defect has been reported in patients with seborrheic dermatitis and eczema is characterized by a defective skin barrier.^24^ The fact that seborrheic dermatitis peaks in infancy could lead to confounding when a diagnosis of eczema is made.^24^ This is coupled with the fact that patients of color suffer a higher burden of seborrheic dermatitis.^25^

Petroleum-based moisturizers, particularly milking jelly, were the most favored among patients. It is peculiar that an agrovet product is popularly used as a skincare product. Dairy farming is a common economic activity in Kiambu County.^26^ Due to affordability, milking jelly has become a favorite substitute for moisturizers.^13^ A randomized controlled trial showed that refined petroleum-based products are effective in the management of atopic eczema.^27^ However, milking jelly is unrefined petroleum which contains polycyclic aromatic hydrocarbons and have been found to have toxic effects on human health.^28^ Furthermore, a study found that high dermal exposure to polycyclic aromatic hydrocarbons precedes skin cancer,^29^ and petrolatum products have been shown to be photosensitizing.^30^ Given that Kenya is located along the equator and the sun is present all year through (coupled with the likelihood that the usage of this product is likely higher), this is alarming.

Thirteen percent of the patients diagnosed with eczema used emollient creams on presentation to the dermatologist which is correct practice. One patient was using a potent topical gluccocorticoid (betamethasone) as a moisturizer. This is a harmful practice as unsupervised use of topical glucocorticoids can lead to harmful effects.^21^^,^^31^ 11% of the patients in the present study did not use a moisturizer, and this presents a gap in the maintenance of a skin barrier.^1^^,^^12^^,^^32^

Another interesting finding was the use of multipurpose bar soap to bathe by 39% of the patients. For barrier integrity, gentle soaps with neutral pH that are devoid of fragrance or preservatives, and have a minimal defatting activity are encouraged.^32^ Multipurpose bar soaps have an alkaline pH, contain fragrance (parfum, coumarin, butylphenyl methylpropional), sodium lauryl sulfate, and preservatives.^33^ This makes them detrimental for eczema. Almost 10% of the patients used medicated soaps. Use of antimicrobial soaps is not advised as it encourages dysbiosis which further dysregulates the skin’s immune response^34^ which, in eczema is known to be dysregulated.^35^

The geospatial clustering in the present study revealed that the majority of the patients with eczema lived in urban areas. This agrees with existing literature.^1^^,^^36^ Hotspots were located around the town centers, the brightest being in Kiambu town where KL5H is located. Most of the patients lived around Kiambu town with a concentric fade as patients resided away from the facility. This suggests that accessibility plays a role in seeking care for eczema.^15^ Environmental factors in urban areas, such as air pollutants, have been found to have a significant impact on the increasing prevalence of eczema in Africa.^37^^,^^38^ In the present study, mapping did not reveal hotspots in the rural agricultural areas that use agrochemicals.

It is important to note that eczema is an umbrella term with the most common form being atopic dermatitis. This paper did not consider various sub types of eczema due to difficulty in accessing specific records. This could lead to an apparently high prevalence. It would be recommended that a standardized data recording tool to be developed for collecting dermatological data.

In conclusion, the prevalence of eczema at KL5H, Kiambu County, Kenya, is found to be 25.5%, predominantly concentrated in urban areas. The study highlights the prevalent use of non-conventional skincare products among patients, indicating a need for further investigation into their impact. Difficulties to access affordable skincare products and patient education on skincare selection will be crucial to improve the local situation.

## Data availability statement

The data used in this study is available in the Kiambu Health department repository and on request to the author.

## Conflicts of interest

None disclosed.
